# Magnetic Resonance Imaging–Based Assessment of Breast Cancer–Related Lymphoedema Tissue Composition

**DOI:** 10.1097/RLI.0000000000000386

**Published:** 2017-05-01

**Authors:** Marco Borri, Kristiana D. Gordon, Julie C. Hughes, Erica D. Scurr, Dow-Mu Koh, Martin O. Leach, Peter S. Mortimer, Maria A. Schmidt

**Affiliations:** From the *Cancer Research UK Cancer Imaging Centre, The Royal Marsden NHS Foundation Trust and The Institute of Cancer Research; †Cardiac and Vascular Sciences, St George's University of London; and ‡Skin Unit, The Royal Marsden NHS Foundation Trust, London, United Kingdom.

**Keywords:** breast cancer–related lymphoedema, tissue composition analysis, image segmentation, magnetic resonance imaging

## Abstract

The aim of this study was to propose a magnetic resonance imaging acquisition and analysis protocol that uses image segmentation to measure and depict fluid, fat, and muscle volumes in breast cancer–related lymphoedema (BCRL). This study also aims to compare affected and control (unaffected) arms of patients with diagnosed BCRL, providing an analysis of both the volume and the distribution of the different tissue components.

**Materials and Methods:**

The entire arm was imaged with a fluid-sensitive STIR and a 2-point 3-dimensional T1W gradient-echo–based Dixon sequences, acquired in sagittal orientation and covering the same imaging volume. An automated image postprocessing procedure was developed to simultaneously (1) contour the external volume of the arm and the muscle fascia, allowing separation of the epifacial and subfascial volumes; and to (2) separate the voxels belonging to the muscle, fat, and fluid components. The total, subfascial, epifascial, muscle (subfascial), fluid (epifascial), and fat (epifascial) volumes were measured in 13 patients with unilateral BCRL. Affected versus unaffected volumes were compared using a 2-tailed paired *t* test; a value of *P* < 0.05 was considered to be significant. Pearson correlation was used to investigate the linear relationship between fat and fluid excess volumes. The distribution of fluid, fat, and epifascial excess volumes (affected minus unaffected) along the arm was also evaluated using dedicated tissue composition maps.

**Results:**

Total arm, epifascial, epifascial fluid, and epifascial fat volumes were significantly different (*P* < 0.0005), with greater volume in the affected arms. The increase in epifascial volume (globally, 94% of the excess volume) constituted the bulk of the lymphoedematous swelling, with fat comprising the main component. The total fat excess volume summed over all patients was 2.1 times that of fluid. Furthermore, fat and fluid excess volumes were linearly correlated (Pearson *r* = 0.75), with the fat excess volume being greater than the fluid in 11 subjects. Differences in muscle compartment volume between affected and unaffected arms were not statistically significant, and contributed only 6% to the total excess volume. Considering the distribution of the different tissue excess volumes, fluid accumulated prevalently around the elbow, with substantial involvement of the upper arm in only 3 cases. Fat excess volume was generally greater in the upper arm; however, the relative increase in epifascial volume, which considers the total swelling relative to the original size of the arm, was in 9 cases maximal within the forearm.

**Conclusions:**

Our measurements indicate that excess of fat within the epifascial layer was the main contributor to the swelling, even when a substantial accumulation of fluid was present. The proposed approach could be used to monitor how the internal components of BCRL evolve after presentation, to stratify patients for treatment, and to objectively assess treatment response. This methodology provides quantitative metrics not currently available during the standard clinical assessment of BCRL and shows potential for implementation in clinical practice.

Breast cancer–related lymphoedema (BCRL) is a chronic swelling of the arm, which develops in approximately 20% of women after breast cancer treatment.^1^ A defining characteristic of BCRL is the accumulation of both interstitial fluid and fat within the arm, which causes both physical and psychological morbidity.^2^ The buildup of protein-rich fluid in the interstitium (edema) is caused by impaired lymphatic transport. However, the mechanisms leading to the abnormal deposition of fat are not fully understood and the links between the lymphatic system and adiposity are still under investigation.^3^ Adipose tissue hypertrophy is likely to be promoted by the inflammatory response triggered by the chronic lymph stasis.^4^ Furthermore, it has been hypothesized that the lymph itself might contain factors that stimulate fat cell differentiation and growth.^5^ The ratio of fat and fluid varies greatly between lymphoedematous arms, yet first-line treatment for BCRL addresses only the fluid, not the fat. Compression and drainage massage attempt to reduce the excess volume by enhancing fluid clearance.^6^ For chronic lymphoedema, liposuction is proposed as a possible intervention.^7^

Quantification of the volume, spatial distribution, and prevalence of the different lymphoedematous tissue components could greatly improve patient selection for optimal treatment. However, standard assessment of lymphoedema is currently limited to a measurement of the size of the affected arm relative to the unaffected arm performed with different methods (circumferential tape measurements along the length of the arm, water displacement, and optical methods [Perometer]).^8^ Because these do not characterize the internal composition of the swelling, prevalence of fluid or fat is typically inferred by manual assessment of pitting. Standard ultrasound imaging can be used to differentiate fat and fluid within the subcutis, but quantitative assessments performed with this technique are limited to a measurement of the thickness of the dermal and subcutaneous layers.^9^ Measurements of percentage tissue composition can be obtained with dual-energy x-ray absorptiometry (DXA) or bioelectric impedance analysis.^10^ However, these techniques do not directly provide the anatomical distribution of the different tissues, and the measurements of composition require assumptions about x-ray attenuation and impedance properties and pathways. Currently, there are no means of simultaneously visualizing and quantifying lymphoedema tissue composition used routinely.

Magnetic resonance imaging (MRI) can produce 3-dimensional (3D) high-resolution images of the internal anatomy of the arm and can be optimized to differentiate various tissue components. Dixon techniques^11^ use images acquired with different echo times to separate fat from the other tissues and are now available in commercial MRI scanners. Heavily T2-weighted sequences are sensitive to fluids^12^ and are commonly used for the visualization of edema. Within the clinical assessment of lymphoedema, these techniques have been used to evaluate single tissue components (edema^13^ or fat^14^) separately.

This article describes an MRI acquisition and analysis protocol that combines fluid- and fat-sensitive sequences to provide maps of lymphoedema tissue composition together with quantitative metrics. This methodology uses fully automated image segmentation to both visualize the distribution of different tissue component within the arm and measure their volume. The proposed technique was used to compare affected and unaffected arms in a cohort of 13 patients with unilateral BCRL.

## MATERIALS AND METHODS

### MRI Protocol

Images of both the affected and the unaffected arms were acquired at 1.5 T (MAGNETOM Aera; Siemens AG, Erlangen, Germany) in separate sessions. The compression garment, if worn, was removed. The patient was positioned supine, with the examined arm extended along the body. The patient's torso was then rotated by 45 degrees, bringing the arm toward the center of the magnet. The patient's back leaned against the side of the magnet's bore, and additional cushions helped to maintain a comfortable and still position. The arm lay palm down on the surface of the bed and was imaged in 3 stations, covering the anatomy from the hand to the axilla with 3 partially overlapping volumes (each with a field of view [FOV] = 300 mm in the superior/inferior direction). The following sequences were acquired in sagittal orientation:

S1, a fluid-sensitive 2-dimensional short-inversion-time inversion-recovery (STIR, TR = 5544 milliseconds, TE = 121 milliseconds, TI = 160 milliseconds, voxel size = 1.2 × 1.2 × 4 mm^3^, acquisition time per volume = 68 seconds).
S2, a 2-point 3D T1W gradient-echo–based Dixon sequence (TR = 12.50 milliseconds, TE = 2.34, 4.77 milliseconds, FA = 12 degrees, voxel size = 1 × 1 × 4 mm^3^, acquisition time per volume = 47 seconds).

The above sequences covered the same total imaging volume (224 × 786 × 160 mm^3^); the scanner software composed the 3 single volumes to form a combined volume. Dixon water and fat images were reconstructed from sequence S2 directly by the scanner. During the reconstruction process, a volumetric inhomogeneity correction was applied.

### Image Processing

An automated segmentation workflow was developed in IDL (version 8.2; Exelis Visual Information Solutions, Boulder, CO). This image postprocessing procedure was designed to simultaneously perform 2 operations: (1) contour the external volume of the arm and the muscle fascia, allowing separation of the epifacial (above the fascia) and subfascial (below the fascia) volumes; and (2), within the previously obtained arm volumes, separate the voxels belonging to the muscle, fat, and fluid components.

**FIGURE 1:**
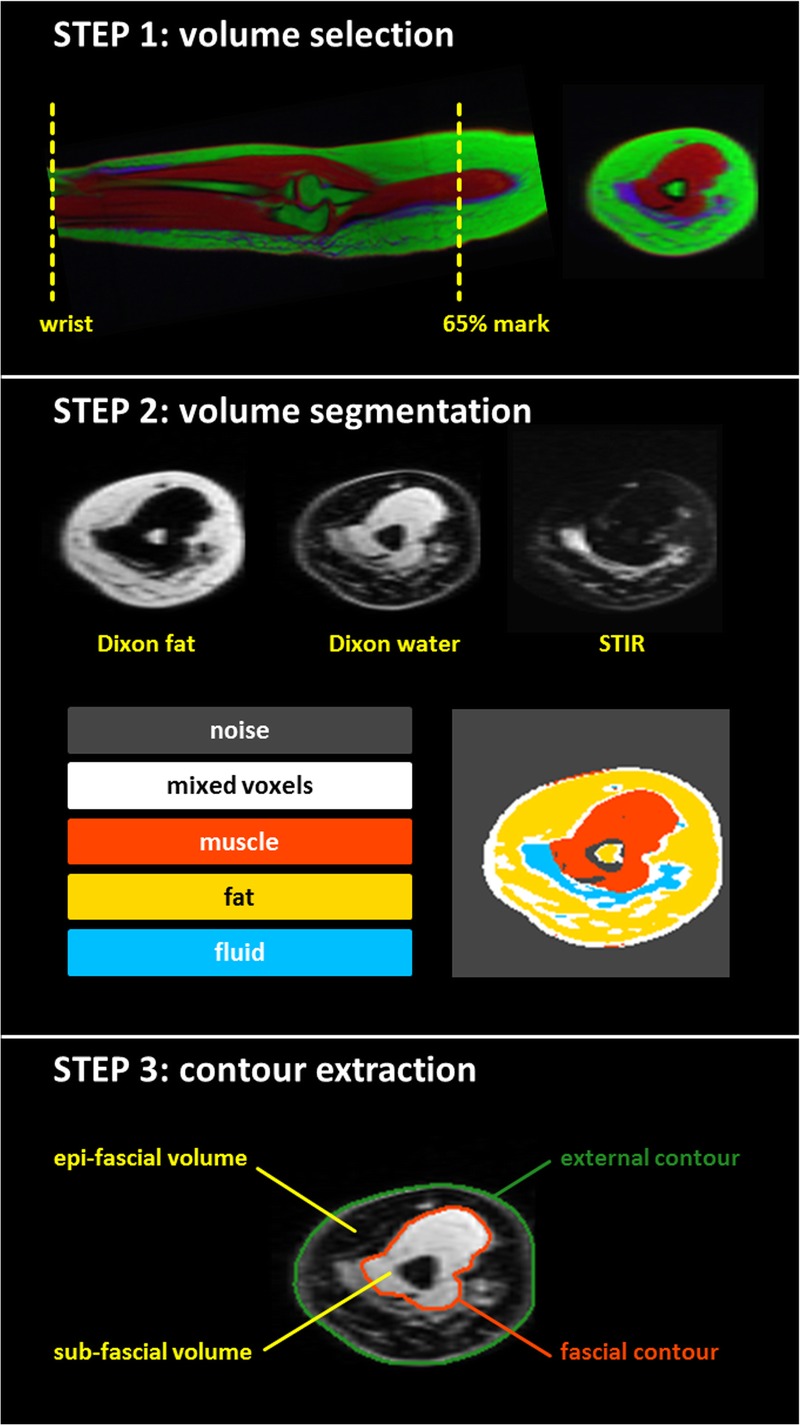
Step 1: Color representation of the overlapped Dixon water (red), Dixon fat (green), and STIR (blue) images. The sagittal (longitudinal) view displays the portion of the arm included in the analysis—between the wrist and the 65% mark (65% of the distance between the elbow and the shoulder tip). The transversal (cross-sectional) view shows how different tissue components (muscle, fat, and fluid) are separated into different images (red, green, and blue images, respectively) on a representative slice. Step 2: Separated Dixon fat, Dixon water, and STIR transversal images, and segmentation map of a representative slice. The k-means algorithm (k = 5) is used to segment the arm volume. Step 3: Fascial and external contours on a representative slice. These encompass, respectively, the subfacial and total arm volumes. The epifascial volume is the volume between the 2 contours.

The workflow is composed of 3 main steps (Fig. 1):

1. Volume Selection
The 3 matched volumes are displayed with 1 × 1 × 1 mm^3^ voxel size and reformatted in transaxial orientation to obtain a series of cross-sectional slices of the arm. A standardized portion of each arm is selected for the analysis. This includes all slices between 2 anatomical landmarks, the wrist (distal radioulnar joint) and the 65% upper arm mark (65% of the distance between the elbow and the shoulder tip).^15^
2. Volume Segmentation
The 3D feature space formed by the voxel intensities of the Dixon water, Dixon fat, and STIR volumes is partitioned into 5 clusters using a k-means algorithm (the code incorporated the implementation from the software package CCHIPS^16^). While the Dixon fat image isolates the fat component, the Dixon water image contains the muscle and the other tissues, and the STIR image selectively depicts the fluid (step 2, Fig. 1). The segmentation process is applied to the combined images, and as a result, muscle, fat, and fluid voxels belong to 3 distinct clusters (red, yellow, and blue clusters in Fig. 1). The k-mean algorithm is initialized with k = 5 and assigns to the 2 additional clusters: (1) voxels contributing no signal (image noise, gray cluster) and (2) voxels with mixed composition at the tissue interfaces, including connective or fibrotic tissue (white cluster).
3. Volume Extraction
The program scrolls through each slice and creates 2 separate masks, containing the entire cross-section of the arm (noise excluded) and the muscle, respectively. Erosion/dilation and triangulation algorithms from the IDL library are then used to automatically extract the external and fascial contours from the masks.^17^ The muscle and the other subfascial tissue components are contained within the fascial contour. Subfacial and total arm volumes are encompassed by the fascial and external contours, respectively, whereas the volume between the 2 contours represents the epifascial volume.

### Clinical Measurements

#### Subjects

Both the affected and the unaffected arms of 13 patients with diagnosed unilateral arm lymphoedema after breast cancer treatment were measured with this technique. All patients were adult women who gave written informed consent as part of a prospective study approved by the National Research Ethics Service. Patient demographics and relevant clinical data are reported in Table 1, specifying the arm affected by lymphoedema and the arm predominantly used (dominant).

**TABLE 1:**
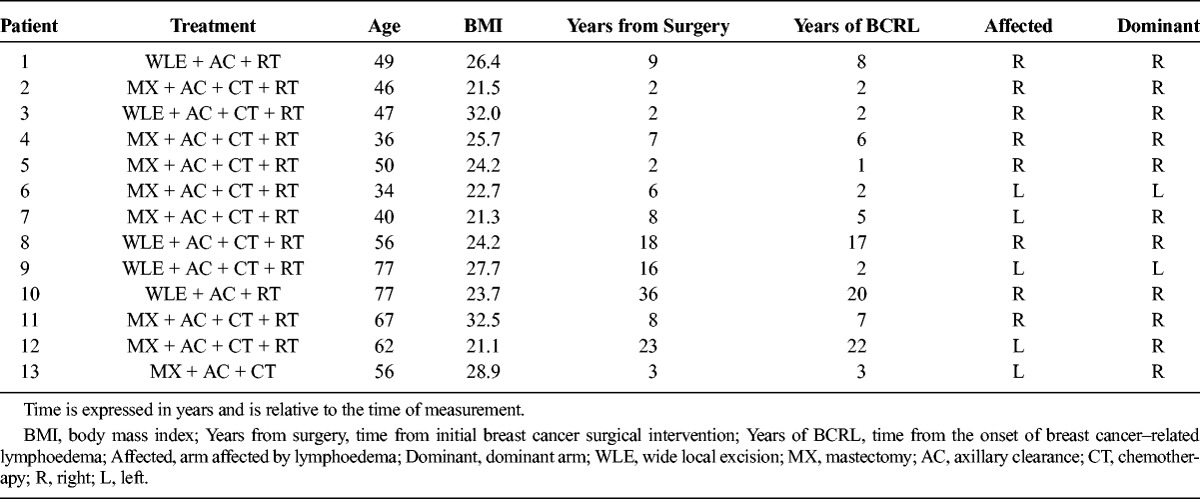
Patient Demographics and Relevant Clinical Data

#### Volume Measurement

Muscle, fat, and fluid subvolumes can be computed by counting the respective number of voxels (1 voxel = 1 mm^3^ = 0.001 mL) within the volumes segmented with the image postprocessing procedure. The following volumes were extracted: total arm, subfascial, epifascial, muscle (subfascial), fluid (epifascial), and fat (epifascial).

#### Volume Visualization

**FIGURE 2:**
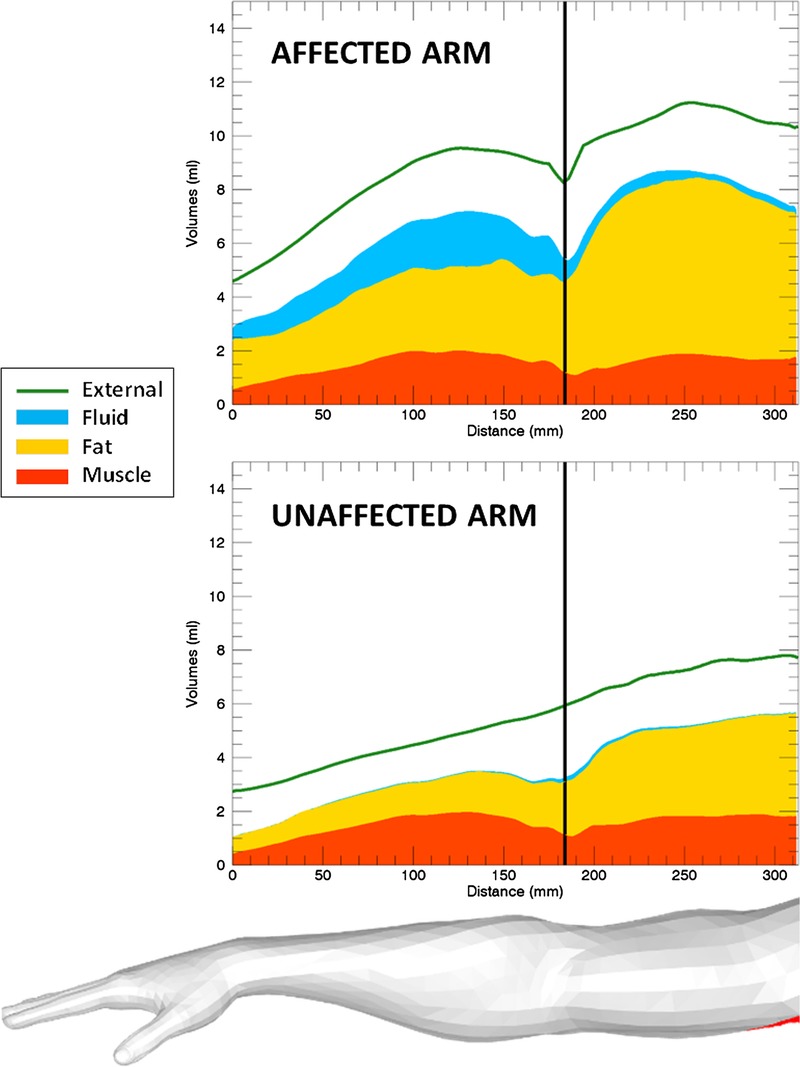
Longitudinal plots of the different tissue volumes within the affected and unaffected arms of an example patient (patient 10): muscle (red), epifascial fat (yellow), epifascial fluid (blue), and total (external, green line).

**FIGURE 3:**
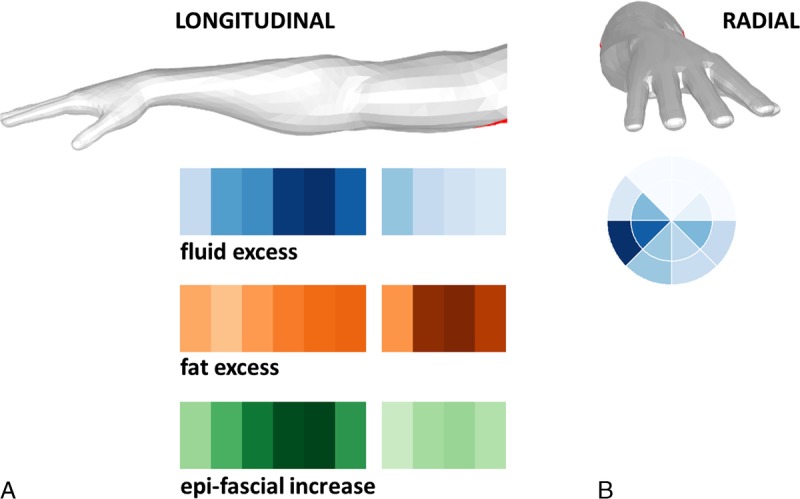
Intensity maps of normalized tissue excess volumes in an example patient (patient 10). A, Image shows longitudinal intensity maps of the distribution of tissue excess along the arm for the 3 tissue components. For fluid and fat, the excess volume is measured as the difference between affected and unaffected volumes, whereas the epifascial increase is measured as the difference in volume as a percentage of the unaffected volume. Darker colors indicate greater values and are normalized to the peak values of each measure—for this patient, the peak values are 60 mL (fluid), 90 mL (fat), and 130% (epifascial increase). B, Image shows the radial intensity map, which gives the distribution of fluid excess in different segments within the epifascial volume, summed over the longitudinal extent of the arm. The external segments represent the layer below the skin, the internal segments the layer above the muscle.

Three different graphical representations (Figs. 2, 3) are used to visualize the distribution of the tissue components within the arm:

##### Longitudinal Volume Plot

Cumulative tissue volumes are plotted along the length of the arm (Fig. 2). Different colors are assigned to different tissue components: muscle (red), fat (yellow), and fluid (blue). The total volume is represented by the green line. The affected and unaffected arm plots can be directly compared, as both contain the same number of slices and the superior/inferior position is matched at the elbow (indicated by the vertical black line).

##### Longitudinal Intensity Map

These graphical representations (Fig. 3A) are used to describe the distribution of tissue excess (affected minus unaffected volume) along the arm. The forearm and the upper arm are arbitrarily divided into 6 and 4 segments, respectively, and for each segment the excess volume of fluid and fat (affected minus unaffected) and the epifascial increase (difference in volume as a percentage of the unaffected volume) are computed and displayed as intensity maps (Fig. 3A). Darker color (blue for fluid, orange for fat, and green for epifascial) indicates greater volume. The color scale is normalized to the peak value within each patient's arm and is therefore not consistent across patients.

##### Radial Intensity Map

Similarly to the previous graphical representations, these intensity maps visualize where the fluid accumulates segmentally around the arm (Fig. 3B). The epifascial volume is divided into 2 layers (external and internal) by computing the midcontour equidistant from the external and fascial arm contours. Each arm cross-section is also divided radially into 8 portions by tracing the horizontal, vertical, and 45-degree-angled lines through the center of mass of the subfascial volume (Fig. 3B). This allows the epifascial volume to be partitioned into 16 segments (8 external and 8 internal). An intensity map is used to visualize the radial distribution of the fluid excess volume, summed over the longitudinal extent of the arm. The external segments represent the layer below the skin; the internal ones represent the layer above the muscle. This graphical representation offers a transversal view of the arm, as indicated by the rendered arm model in Figure 3B. To allow comparison between patients, the side of the arm facing the torso is always represented on the right side of the map, independently of which arm is imaged.

#### Statistical Analysis

Statistical descriptions, tests, correlations, and linear regressions were performed with R (version 3.3.1; The R Foundation for Statistical Computing, Vienna, Austria).

##### Affected Versus Unaffected

All the sets of volumes were tested for normality using the Shapiro-Wilk test. Affected versus unaffected volumes were compared using a 2-tailed paired *t* test; a value of *P* < 0.05 was considered to be significant.

##### Fat Versus Fluid

Pearson correlation was used to investigate the linear relationship between different sets of volumes.

##### Correlation With Clinical Data

Correlation between relative excess tissue volumes (affected minus unaffected) and clinical parameters was also investigated. The following excess volumes were included in this analysis: epifascial, fat, and fluid. These were divided by the epifascial volume of the unaffected arm to produce a set of relative excess volume measurements, where differences in arm size across patients are normalized. The relative excess volumes were correlated with age, body mass index, time from breast cancer treatment, and time from BCRL development. Because the clinical parameters might not be normally distributed, the nonparametric Spearman rank correlation test was adopted (2-tailed, *P* < 0.05).

## RESULTS

### 

#### Volume Measurement

Considering the whole cohort, the increase in epifascial volume (globally, 94% of the excess volume) constituted the bulk swelling. Within the epifascial volume, fat was the main component of the swelling (the total fat excess volume summed over all patients was 2.1 times that of fluid). Table 2 directly compares the different tissue volume measurements in affected and control (unaffected) arms. Percentages in brackets indicate the percentage volume increase (or decrease) of the affected volume relative to the corresponding unaffected volume, according to the formula (affected − unaffected)/unaffected.

**TABLE 2:**
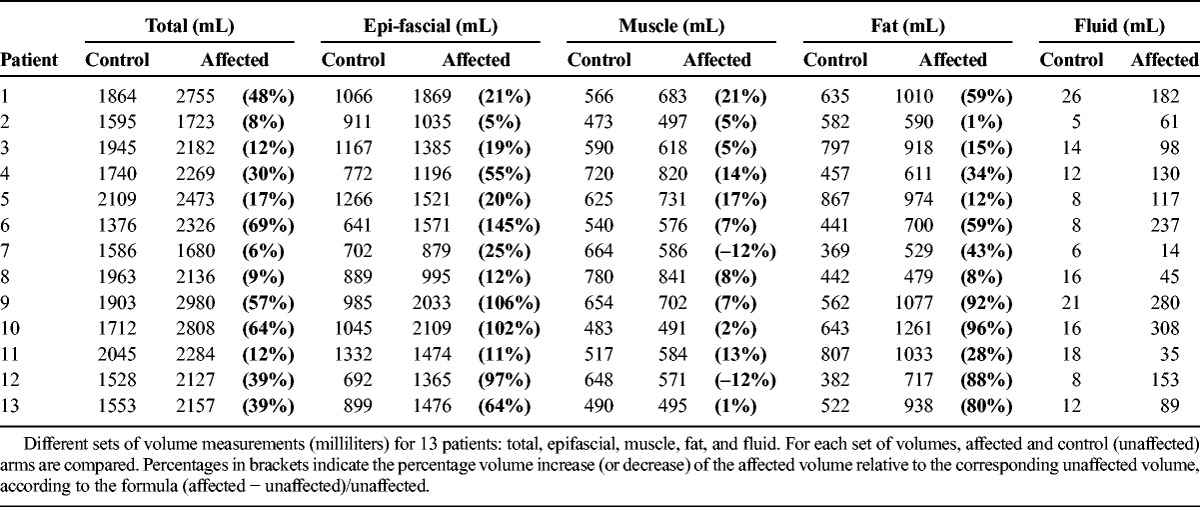
Different Sets of Volume Measurements for 13 Patients

##### Affected Versus Unaffected

The following sets of volumes were considered: total, subfascial, epifascial, muscle, fluid, and fat. All the sets passed the normality test. Total, epifascial, fluid, and fat volumes were significantly different (*P* < 0.0005) between affected and unaffected arms, with greater volume in the affected arms (Table 3). The muscle volume was not significantly different between the 2 arms. In 10 of 13 patients, the affected arm coincided with the dominant arm; after dividing the muscle volumes into dominant and nondominant, dominant muscles were found to be significantly greater (*P* < 0.0008). Muscle volume differences are likely to be influenced both by arm use and, possibly, by lymphoedema-induced hypertrophy. Nevertheless, in this cohort of patients, the subfascial swelling contributed in total only 6% to the excess volume.

**TABLE 3:**
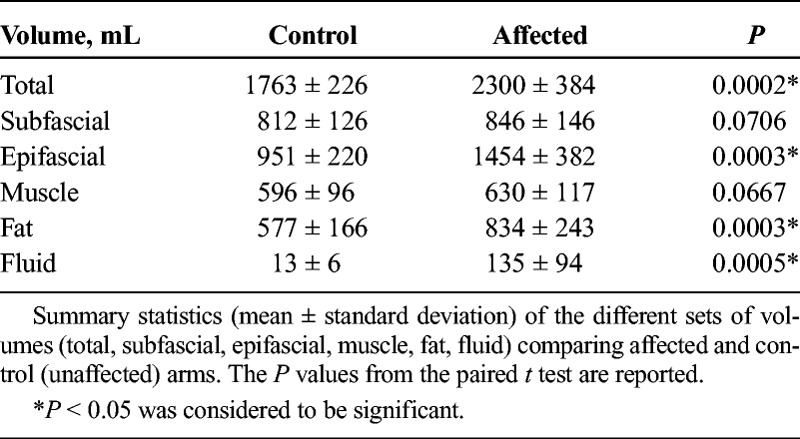
Summary Statistics of the Different Sets of Volumes

##### Fat Versus Fluid

**FIGURE 4:**
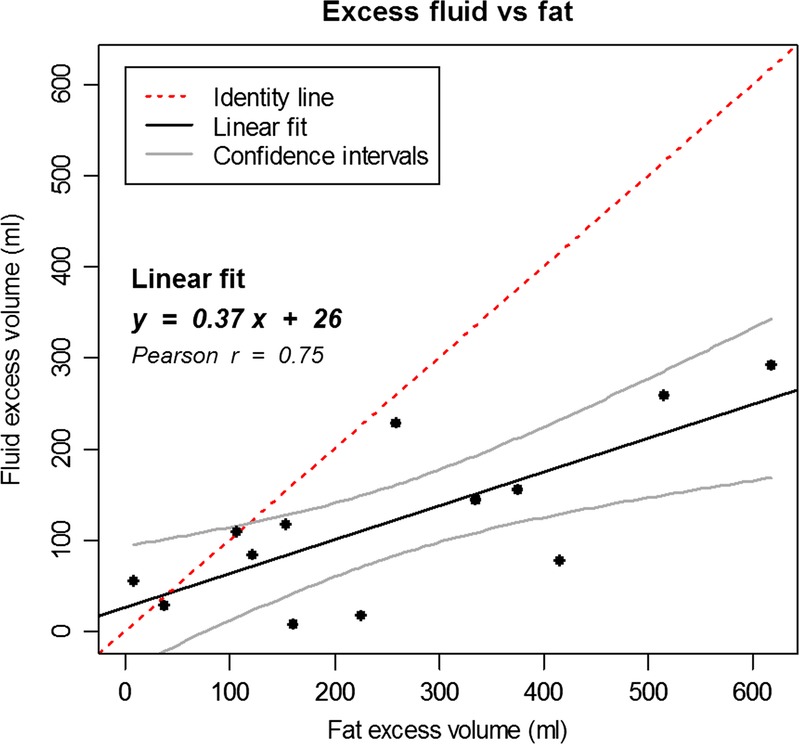
Correlation between fat and fluid excess volumes. The continuous black line represents the linear fit (equation reported on the graph), whereas the continuous gray lines represent the 95% confidence intervals. The correlation is significant (*P* = 0.003, Pearson correlation coefficient reported on the graph). Most points fall below the identity line (dotted line).

Fat and fluid excess volumes were linearly correlated in the cohort of patients analyzed (Fig. 4); in the majority of the cases, the excess of fluid can be expected to be less than half the excess of fat. Figure 4 also shows that some patients did not necessarily follow this linear trend and, in proportion, had predominant accumulation of 1 of the 2 components. Nevertheless, in absolute terms, the excess of fat was greater in 11 subjects (all the points falling below the identity line).

##### Correlation With Clinical Data

The relative excess volume of fat had a moderate but significant positive correlation with the time from breast cancer surgery (Spearman *r* = 0.566, *P* = 0.047).

#### Volume Visualization

##### Single Patient

The previous results describe the differences between affected and unaffected sets of volumes within the patient cohort. The tissue volume measurements in Table 2 combined with the graphical representations in Figures 2 and 3 can be used by a clinician to describe how BCRL affects a patient's arm. While the longitudinal plots in Figure 2 display the distribution different tissue components along the arm and allow a direct comparison of affected and unaffected arms, in Figure 3, the intensity maps visualize the distribution of tissue excess (affected minus unaffected volume) along the arm (Fig. 3A) and segmentally around the arm (Fig. 3B).

Figures 2 and 3 refer to patient 10. Considering this patient as an example, the affected arm is 64% larger than the unaffected arm (Table 2). We observe accumulation of both fat and fluid external to the muscle in the affected arm. The muscle volumes are similar in the 2 arms (2% difference, compatible with differences due to hand dominance and arm use). The fat volume is almost doubled in the affected arm (96% higher than the unaffected arm), and fat is therefore the main component of the swelling (relative percentages are 68% fat and 32% fluid). Nevertheless, fluid buildup is substantial and sits predominantly below the elbow (Figs. 2, 3A). Considering the distribution around the arm, the fluid accumulates at the bottom of the external side of the arm, with a greater deposition immediately below the skin and, to a lesser extent, above the muscle (Fig. 3B). In longitudinal views of the arm (Figs. 2 and 3A), fat accumulates along the whole arm, and particularly above the elbow (a region which is physiologically fattier). However, in relative terms, the swelling is more prominent in the forearm (Fig. 3A, green map), where both fat and fluid accumulate. For this patient, compression should focus on this area to enhance fluid flow, whereas compression above the elbow is ineffective due to the prevalence of fat, which cannot be compressed.

##### Patient Cohort

**FIGURE 5:**
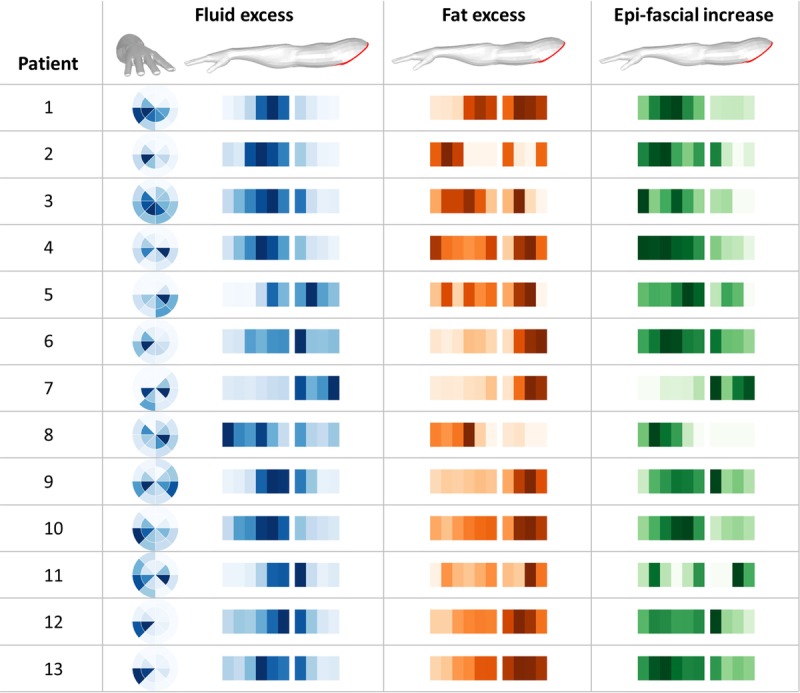
Array of radial and longitudinal intensity maps for 13 patients, where each row represents a single patient. For each measure, the color intensities are normalized to the peak value in each patient, see Figure 3 for a detailed description of these graphical representations.

Figure 5 offers a bird's-eye view, over the whole patient cohort, of the hot spots where fat and fluid accumulate within the arm (blue and orange maps, respectively), and where the arm volume increases the most (green map). The graphical representations described in Figure 3 are here displayed in a vertical array where each row represents a single patient. Figure 5 shows that, although the distributions of tissue excess have a degree of heterogeneity across patients, some common patterns can be found. Fluid accumulated predominantly in the forearm and around the elbow (longitudinal blue intensity maps), with substantial involvement of the upper arm in only 3 of 13 cases. The radial intensity maps indicate that fluid did not distribute uniformly around the arm, but sat preferentially in specific areas, with patterns that were heterogeneous across patients. Regarding the distribution of fat (orange intensity maps), in absolute terms, the excess volume was generally greater in the upper arm, an area that physiologically contains more fat. However, the relative increase in epifascial volume (green intensity maps), which considers the total swelling relative to the original size of the arm, was maximal within the forearm in 9 of 13 cases. This indicates predominant involvement of the distal portions of the arm, further away from the original axillary damage.

## DISCUSSION

Breast cancer–related lymphoedema remains a common problem and a difficult treatment challenge. First-line BCRL treatment involves massage, compression, exercise, and attempts to addresses the fluid excess, but cannot remove fat. This article presents an MRI-based methodology to describe pathological accumulations of both fat and fluid in a quantitative and 3D way. This type of information is not currently available during the standard clinical assessment of BCRL and could allow the delivery of a personalized treatment. Tissue volume measurements as proposed here may help to identify those patients who would still benefit from standard decongestive treatment or those suitable for liposuction, where fat dominates the swelling. Furthermore, the quantitative metrics introduced in this work could guide treatment delivery and could be used to improve the compression strategy (by avoiding additional compression in fatty areas) or to direct drainage massage toward the area where the fluid sits. Finally, longitudinal measurements would provide the possibility of quantitatively assessing how the different tissue components respond to the treatment applied.

In this work, we have used the proposed methodology to assess a pilot cohort of BCRL patients. In this cohort, some patients have experienced lymphoedema for years and others were diagnosed more recently. Even within this heterogeneous group, the volume of fat was predominant in most cases. This finding is of clinical interest, as it suggests that fat, which cannot be removed by first-line treatment, might be the dominant component of BCRL. This indicates that if treatment of BCRL is to improve greater consideration needs to be given to the accumulation of fat; in cases where this is predominant, liposuction should be considered at an earlier stage of management.

Within the clinical assessment of body tissue composition, DXA^10^ and whole-body MRI^18,19^ represent basic options for the quantification of fat and muscle volumes. However, these 2 techniques do not currently detect the presence of fluid and do not provide detailed information on tissue distribution in specific anatomical regions. Magnetic resonance imaging has the advantage of not requiring ionizing radiation and being intrinsically a volumetric technique. The MRI-based methodology proposed in this article requires a 6-minute-long scan and including the positioning of the patient, a 15- to 20-minute scanner slot should be allocated. This is considered feasible for the adoption of this examination in clinical practice. Once the image volumes are prepared and the anatomy from the wrist to the 65% upper arm mark is selected (5 minutes), the algorithm uses less than a minute to analyze the images and automatically extract and visualize the results. In terms of time resources, this procedure lasts as long as a Perometer or tape measurement (15–20 minutes). However, with this approach the clinical user can obtain, in addition to the total volume of the arm, automated measurements of both internal anatomical subvolumes and tissue composition. Compared with DXA or bioelectrical impedance analysis, an approach based on 3D MRI of different tissues offers several advantages: (1) fat, fluid, and muscle volumes can be measured simultaneously; (2) as the various tissue volumes are reconstructed in 3D, volume measurements are direct and not dependent on a signal model; (3) differences in tissue volume can be visually assessed; and (4) as the different tissue volumes are segmented and mapped, automated image postprocessing can be used to produce a wide range of spatially localized measurements.

The proposed methodology can assess the accumulation of fluid; this is detected by MRI as a hyperintense signal on heavily T2-weighted STIR sequences.^12^ It has been shown that different visual patterns can be found in BCRL; these vary from widespread areas of uniform hyperintense signal (indicating bulk accumulation of fluid) to the characteristic honeycomb pattern (a trabecular pattern where fluid is trapped between adipose of fibrotic tissue cells).^13^ The technique described in this article is sensitive to macroscopic presence of fluid and can depict fluid if this is the predominant species within the dimensions of a voxel. This technique uses 3 MRI images (Dixon water, Dixon fat, and STIR) to separate the tissue components of interest (muscle, fat, and fluid). The voxel intensities from the 3 images form defined clusters of data within the feature space where the segmentation operates, and therefore the algorithm behaves consistently across different arms, whether they are affected or not by BCRL. While the Dixon fat image isolates the fat signal, the combination of STIR and Dixon water images allows optimal separation between the fluid and muscle components (Fig. 1, step 2), which are often anatomically close. This is important for the effective delineation of the fascial contour, which divides the epifascial and the subfascial volumes. The segmentation and contouring workflow, applying these robust principles, successfully processed 26 arms.

Our measurements indicate that the bulk of swelling lies within the epifascial volume (the layer between the skin and the muscle, surrounded by connective tissue), where fat and fluid accumulate. In the cohort of patients analyzed, the fat excess volume was substantially greater than the fluid in most patients and was therefore the main contributor to the epifascial swelling. Differences in muscle volume between the 2 arms did not substantially contribute to the excess volume. The total volume of the muscle compartment summed over all patients was only 6% larger in the affected arms, and this result is likely to be influenced by arm use. This volume increase is substantially lower than the 47% increase reported by Brorson et al,^20^ measured by DXA, and therefore, in our cohort, we did not observe muscle hypertrophy due to advanced lymphoedema.

In our results, we found confirmation that lymphoedema does not affect the arm uniformly. The possibility to visualize where fat and fluid accumulate allows to identify the portions of the arm where 1 of the 2 components is prevalent. The studies confirm the clinical impression that fluid collects mainly around the forearm and elbow region, whereas fat mainly contributes to upper arm swelling (Fig. 5). The reason for this is not known. This methodology could help understand the temporal changes that take place in the evolution of BCRL. Furthermore, this approach could help determine the effects of treatment interventions on the internal composition of a swollen arm.

In summary, the main aim of this article is to present a methodology, based on MRI and automated image postprocessing, which can (1) provide a series of measurements of lymphoedema tissue volumes (Table 2) and (2) display the distribution of different tissue components within the arm, using intuitive visual representations (Figs. 2, 3). An approach to BCRL management which uses the metrics introduced in this article could guide the clinician regarding therapeutic approaches, saving time and resources on unnecessary treatment.

This work is subject to some limitations. Our pilot cohort of patients is small and contains subjects with a clinical history of heterogeneous lymphoedema, limiting the extent of our correlations with clinical data. To characterize different and specific breast cancer groups, lymphoedema stages, and management regimes, the proposed methodology should be applied to larger and more homogeneous populations. Furthermore, the proposed 6-minute MRI acquisition protocol is not able to detect the presence of fat within the muscle fibers as performed by Peterson et al,^14^ as this would have required dedicated MRI sequences with greater sensitivity to this type of deposition, longer scanning times, and further image processing. However, this is not the focus of our development, which aims at providing a global quantification and description of the different anatomical and tissue components of the whole arm. Finally, this methodology is not currently optimized to characterize the presence of fibrosis, as the MRI sequences and the segmentation focus predominantly on isolating fluid, fat, and muscle. At this stage, we have prioritized the development of a robust procedure that could readily be implemented using commercial MRI sequence packages.

This quantitative methodology is suitable for longitudinal studies: in future work, we will use it to monitor how the internal components of BCRL evolve after presentation, to stratify patients for treatment, and to objectively assess treatment response, including impact on different tissue components. Magnetic resonance imaging approaches are also used to image lymphatic vessels and characterize lymphatic transport.^21,22^ If information on vessel morphology or local lymphatic failure was available through dedicated imaging, this could be spatially correlated with tissue volume measurements.

In conclusion, with a 15-minute MRI examination and a fully automated postprocessing, the proposed methodology provides a complete assessment of the composition of tissues affected by BCRL. This technique was able to both visualize the distribution of fat, fluid, and muscle within the arm and measure their volume. In the cohort of patients analyzed, excess of fat within the epifascial layer was the main contributor to the swelling, even when the fluid component was substantial. Furthermore, muscle differences between affected and unaffected arms did not contribute markedly to the total excess volume. Changes in tissue composition associated with BCRL were not uniform along the arm; this highlights the relevance of an approach, which can provide information on the spatial distribution of the different tissues. Measurements of tissue volume as proposed here may help the BCRL clinician select the best treatment strategy. Moreover, this image-based approach could guide treatment delivery, for example, by indicating the optimal site for drainage massage or by providing additional structural information to design compression garments. Finally, quantitative characterization of tissue composition by MRI as presented here can be used to objectively evaluate response to different lymphoedema management strategies.
